# Institutional Notes and News

**Published:** 1910-06-04

**Authors:** 


### Institutional Notes and News.

St. Thomas's Hospital was fortunate last year in receiving an anonymous present of £5,000, which made income and expenditure much more nearly to tally than they would have done otherwise. The figures for the year were given in Mr. Wainwright's treasurer's report as income £62,650 and expenditure £63,431. The patients numbered 7,715 in the wards and 83,728 in the out-patient department, and of the latter 1,377 were maternity cases. The new ward for them is to be opened before the autumn. The treasurer congratulated the hospital upon the reduction in the weekly cost of maintenance and in the average total cost of each in-patient; in the first case that cost now is £1 18s. 1½d., in the second the average cost is £7 3s. 7¾d. The *x*-ray department was kept busy with patients, 4,020 persons being treated. It should be added that the daily average number of occupied beds was 521, or twenty-three more than last year, while the number of beds available was 561.

**St. Thomas s Hospital.**

H.S.H. Prince Francis of Teck, who has been appointed recently Chairman of the Weekly Board of the Middlesex Hospital, made an interesting speech in moving the adoption of the report at the Court of Governors. In referring to the late King, the Prince said how deeply touched he would have been if he could have known that the poor ladies in the cancer wing had subscribed their pence and sent a wreath in loyal memory of their great patron. He went on to say that the scheme for the Barnato-Joel Charity had been approved by the Charity Commissioners, and that the site had been cleared. It is hoped that the building, to be begun almost at once, will be finished in a year's time. The Education Committee of the London County Council has been approached, with the result that a certificated lecturer in cookery has been instructing the nurses in cooking for invalids, and this instruction now forms part of the regular curriculum. Another improvement worth notice is that all cases of pulmonary tuberculosis which attend the out-patient department are now notified.

**Middlesex Hospital.**

The Annual Founders' Day Commemoration of the Middlesex Hospital was celebrated by a service in the Hospital Chapel at three o'clock in the afternoon of Thursday, May 26. A new organ has recently been erected in the chapel by private benefactions, and in the course of the service short recitals were given by Mr. F. A. Docker, F.R.A.M., organist and choir-master at St. Andrew's, Wells Street, and Mr. F. H. Stamper, A.R.C.O., assistant organist of the same church. The service was conducted by the Rev. Herbert E. Gunson, M.A., chaplain to the hospital and Cancer Charity, who also preached the sermon. There was a large congregation, which included His Serene Highness Prince Francis of Teck, G.C.V.O., D.S.O., chairman of the Weekly Board of Governors; the Hon Spencer Lyttelton, C.B., vice-president; Sir Richard Douglas Powell, Bart., K.C.V.O.; members of the honorary staff; and many governors and friends. Among the gifts to the chapel during the past year is the marble font of remarkable design. The Chaplain announced that the chapel was entirely free of debt. After the service visitors had the opportunity of inspecting the hospital and the Cancer Charity.

**Founders' Day at the Middlesex Hospital.**

The in-patients at Guy's Hospital continue to increase in number, and no fewer than 8,933 were treated in the past year, which is a record even at this institution. Extensive schemes for the alteration and improvement of the buildings are being considered, and one of them, the new out-patient department, was completed and paid for. Other plans, including separate wards for children, further beds for the special departments, and the rebuilding of the clinical house are hoped to be carried through before long; but certain matters of greater urgency are to be undertaken first. These are a new operation unit, an extension of the Henriette Raphael Nurses' Home, and better residential quarters for certain sections of the staff. A year ago, it will be remembered, an appeal for £60,000 was issued, the sum which these latter improvements were expected to cost, and a 1910 Building and Renovation Fund is now open to receive subscriptions for this express purpose.

**Guy's Hospital.**

The question of a new infirmary for Bradford has agitated for a long time the minds of Bradford people, and a scheme which concerns one of the great industrial centres of the North has vital interest for British hospital workers everywhere. We would recall to our readers the progress which the scheme has made. Last year may be described as the one in which the plan to provide Bradford with an infirmary worthy to stand beside any other provincial hospital of the empire took definite shape. The bold and sensible step was taken of appealing for money on the definite ground that no building should be undertaken till £100,000 was in the bank. Bold policies, if they have brains behind them, always succeed, and the amount of brains in Bradford was demonstrated by the astonishing quickness with which the first £58,000 was subscribed. Mr. James Hill was Lord Mayor of Bradford that year, and every obstacle, bad trade, the thousand-and-one deadeners of public spirit (which Londoners more than provincials, as Sir Dyce Duckworth recently pointed out in The Hospital, know well), were overcome by his enthusiasm. This has inspired the office he held, which is the best proof of its quality, and the succeeding Lord Mayor, Mr. Alderman William Land, carried on the good work so well that a further £10,000 was quickly added. The working men of Bradford, however, have too much public spirit to allow even the leaders of the city to do all the work, and the way in which they have come forward guarantees to the new infirmary not only adequate buildings, but adequate support for their maintenance. The building is only half the battle, and University Hospital is but one example of the fact that a splendid equipment is paralysed without a splendid endowment at its back. The question of a site, never an easy matter, was settled at last, and the thanks of the present Royal Infirmary have been given publicly to the gentleman whose reports on English hospitals have been appearing elsewhere in this newspaper. The site, then, comprises 23 acres, stands 600 feet above the sea-level, and, while outside the city, is in direct communication by tram with every part. The site was purchased from the executors of the late Mr. Isaac Smith, and is known as the Field House Estate, at least by Bradford citizens. Judging from the steady growth of the fund and the genuine public spirit awakened, it should not be very long before the new infirmary stands over the city, a monument of what public enthusiasm and expert knowledge can do.

**The New Infirmary, Bradford.**

